# Properties of Heterochannels Kv(1.1-1.2)_2_ with Mutation T226R in the Kv1.1 Subunit

**DOI:** 10.3390/ijms26199730

**Published:** 2025-10-06

**Authors:** Anastasia A. Ignatova, Anastasia V. Efremenko, Denis V. Abramochkin, Irina Dzhumaniiazova, Ivan I. Shmatin, Mikhail P. Kirpichnikov, Alexey V. Feofanov, Oksana V. Nekrasova

**Affiliations:** 1 Shemyakin-Ovchinnikov Institute of Bioorganic Chemistry, Russian Academy of Sciences, 117997 Moscow, Russia; aignatova_83@mail.ru (A.A.I.); aefr@mail.ru (A.V.E.); shmatinva@gmail.com (I.I.S.); kirpichnikov@inbox.ru (M.P.K.); onekrasova@ibch.ru (O.V.N.); 2 Faculty of Biology, Lomonosov Moscow State University, 119234 Moscow, Russia; abram340@mail.ru (D.V.A.); dzhumaniiazova.irina.2019@post.bio.msu.ru (I.D.); 3 Institute of Physiology, Pirogov Russian National Research Medical University, 117513 Moscow, Russia

**Keywords:** Kv1.2, Kv1.1, mutation in *KCNA1*, episodic ataxia, epilepsy, heterochannel, fluorescent microscopy, patch clamp

## Abstract

Mutation T226R in the Kv1.1 α-subunit of voltage-gated potassium Kv1 channels is associated with episodic ataxia type 1, severe neuromyotonia, and epilepsy. In vitro, this mutation was reported to considerably distort the functioning of homotetrameric channels Kv1.1; however, in the brain, Kv1.1 α-subunits form heterochannels predominantly associating with Kv1.2 α-subunits. Using the patch-clamp technique, fluorescent and Förster resonance energy transfer confocal microscopy, we revealed that heterochannels Kv(1.1(T226R)-1.2)_2_ formed by concatemers Kv1.1(T226R)-Kv1.2 in Neuro-2a cells have significantly slower activation and deactivation rates, and their activation occurs at a much less negative membrane potential compared to channels Kv(1.1-1.2)_2_ formed by concatemers Kv1.1-Kv1.2. This mutation does not noticeably affect the formation of complexes between α-subunits Kv1.1 and Kv1.2, but it does induce a delayed and possibly decreased presentation of heterochannels Kv(1.1(T226R)-1.2)_2_ on the plasma membrane. At the same time, the T226R mutation has a much stronger negative effect on the membrane presentation of homotetrameric Kv1.1 channels. Since heterochannels Kv1.1-Kv1.2 but not homotetrameric channels Kv1.1 are present in the brain, the heterochannels bearing mutation T226R are most likely underlying the pathogenesis of the disease by decreasing the responsiveness of cells to mild membrane depolarization and, thus, increasing the excitability of neurons.

## 1. Introduction

Potassium ion channels, which are characterized by a significant diversity in structural and functional properties, as well as complex distribution within organs and tissues, regulate many cellular processes, such as neuronal excitability [1], muscle contraction [2], glucose homeostasis [3], magnesium reabsorption in kidneys [4], and proliferation and cytokine secretion in immune cells [5]. Considering the functional importance of K^+^ channels, it is not surprising that many mutations leading to their dysfunction are associated with various Mendelian disorders such as type 2 diabetes mellitus [6], cardiac arrhythmias [7], and brain disorders, including behavioral and neuronal diseases [8,9].

A number of mutations found in voltage-gated potassium (Kv) channels are linked to epilepsy, one of the most common brain disorders characterized by a variety of clinical manifestations and pathophysiological mechanisms [8,10]. Pathogenic mutations that are associated with epilepsy and epileptic encephalopathy, as well as with episodic ataxia type 1 (EA1), myokymia, and combinations of these disorders, have been found in the *KCNA1* gene, which encodes the Kv1.1 α-subunit involved in the formation of various voltage-gated potassium Kv1 channels [11,12,13].

Structurally, the Kv1.1 α-subunit, similar to other α-subunits of the Kv1 channel family, consists of six transmembrane helices (S1–S6) and N- and C-terminus cytoplasmic domains. When four α-subunits form a channel, the helices S5 and S6 connected by a P-loop comprise the pore domain, while helices S1–S4 shape a voltage-sensing domain that is responsible for controlling the gating of the channel [14]. EA1 is associated with mutations that are evenly distributed across S1–S6 helices of the Kv1.1 α-subunit, whereas seizures are caused by mutations that are predominantly localized in S2, S3, and the pore domain [11].

In the brain, the Kv1.1 α-subunit is one of the most abundant constituents of Kv1 channels [15,16]. It is expressed in the hippocampus, cerebellum, and neocortex and is primarily localized in the axon initial segments, juxtaparanodal regions of myelinated axons, and axonal terminals [17,18]. Kv1.1 is known as a critical regulator of action potential propagation along the axon [19]. Although Kv1.1 α-subunits can form homotetrameric channels, in the brain, they form heterotetrameric channels predominantly by associating with Kv1.2 and, to a lesser extent, Kv1.4 α-subunits [15,16,20].

In normal physiology, the Kv1.1 α-subunit is important for the repolarization of the membrane during the interval between action potentials, helping to prevent excessive excitation of neurons. The knockout of *KCNA1* leads to increased excitability, which causes frequent spontaneous seizures in mice [21,22]. In contrast, increased ectopic expression of the Kv1.1 channel in demyelinated axons in a mouse model of multiple sclerosis led to a decrease in nerve excitability, which is consistent with the pathophysiology of multiple sclerosis [23].

The focus of our study is a T226R mutation in the Kv1.1 α-subunit, which is localized at the top of the S2 helix in a region that is highly conserved for Kv1 channels. Among the known T226 mutations, T226R is associated with a severe phenotype, including epilepsy [24], whereas T226A/M exhibits milder ataxic symptoms [25,26], and T226K is associated with non-ataxic symptoms [27]. The mutation T226R was revealed in various clinical cases that exhibit EA1 symptoms [24,28] and EA1 with epilepsy [24], as well as other non-ataxic presentations such as severe neuromyotonia and skeletal deformities [28], developmental delay, and periodic muscle spasms [29]. Given the wide spectrum of clinical symptoms, there is no direct correlation between genotype and phenotype in the case of the T226R mutation. This suggests that various factors at the molecular, cellular, and organismal levels may be involved in the pathophysiology of disorders linked to this mutation.

Electrophysiological studies on Kv1.1 channels heterologously expressed in oocytes revealed that the T226R mutation caused a significant reduction in the whole-cell current amplitudes, a shift in the channel opening toward a more positive membrane potential, as well as slower activation and deactivation of the channel [24,30,31]. It was supposed that the T226R mutation impairs the membrane presentation of the channel and possibly negatively affects its assembly [30]. It was found that the mutation effect was reduced when the Kv1.1(T226R) α-subunit formed heterochannels with the unmutated Kv1.1 α-subunit, and it was concluded that the heterozygous genotype may decrease the impact of the T226R mutation on the organism [24,30,31]. However, considering that heterochannels formed by Kv1.1 and Kv1.2 α-subunits dominate in cells of the nervous system over homotetrameric channels Kv1.1 [20], it is necessary to study the effect of the T226R mutation on the function of these heterochannels.

Recently, we have constructed the plasmid that encodes the concatenated dimer of human Kv1.1 and Kv1.2 α-subunits fused with the fluorescent protein mKate2 (K-Kv1.1-1.2) [32]. K-Kv1.1-1.2, expressed in mammalian cells, has been shown to form functional heterochannels, Kv(1.1-1.2)_2_, with a 2:2 stoichiometry of Kv1.1 and Kv1.2 α-subunits [32]. In the present study, we report on the properties of heterochannels Kv(1.1-1.2)_2_ affected by the T226R mutation in the Kv1.1 α-subunit as well as on the effect of this mutation on the membrane presentation of homotetrameric Kv1.1 channels.

## 2. Results

### 2.1. Design of the Protein Constructs

To study the effect of the T226R mutation in the Kv1.1 α-subunit on the properties of heterochannels Kv(1.1-1.2)_2_ and membrane expression of homotetrameric channels Kv1.1, the following chimeric proteins encoded by the corresponding plasmids were used:

K-Kv1.1, the human Kv1.1 α-subunit fused at the N-terminus with the fluorescent protein mKate2 and bearing mutation S369T that increases the membrane presentation of homotetrameric channels Kv1.1 [33];

C-Kv1.2, the human Kv1.2 α-subunit fused at the N-terminus with the fluorescent protein TagCFP and bearing mutation S371T that increases the membrane presentation of homotetrameric channels Kv1.2 [34];

K-Kv1.1m, the K-Kv1.1 protein bearing mutation T226R;

K-Kv1.1-1.2, the concatenated dimer of Kv1.1(S369T) and Kv1.2(S371T) α-subunits that were linked together via the KL linker and fused at the N-terminus with mKate2 [32];

K-Kv1.1m-1.2, the K-Kv1.1-1.2 protein bearing mutation T226R.

### 2.2. Distribution of K-Kv1.1m and K-Kv1.1m-1.2 in Cells

**Figure 1:**
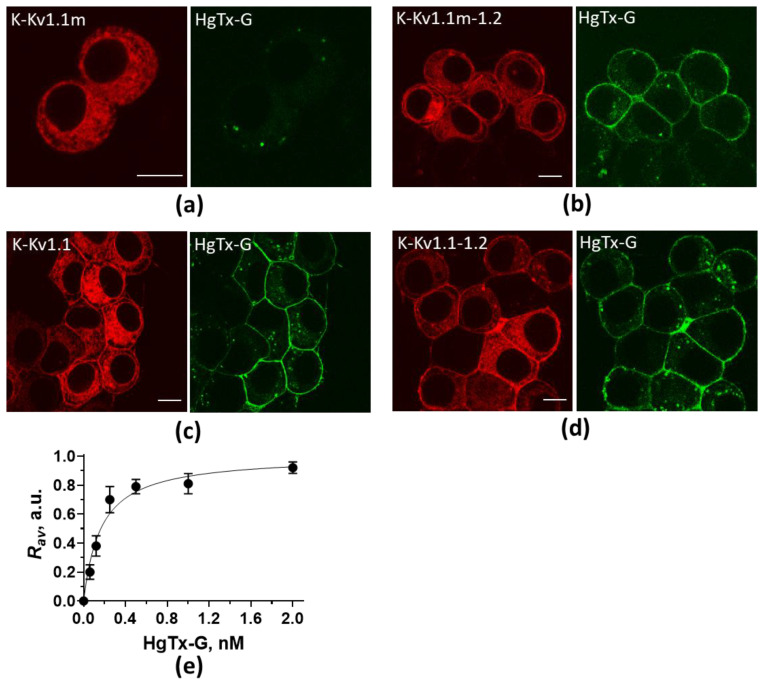
Confocal fluorescent images of intracellular distributions of K-Kv1.1m (**a**), K-Kv1.1m-1.2 (**b**), K-Kv1.1 (**c**), and K-Kv1.1-1.2 (**d**) and staining of cells with HgTx-G. Distributions of expressed protein constructs and HgTx-G are shown in red and green, respectively. Cells were incubated with 2 nM (**a**,**c**) or 0.5 nM (**b**,**d**) HgTx-G for 1 h. The bar is 10 μm. (**e**) Concentration-dependent binding of HgTx-G to heterochannels Kv(1.1m-1.2)_2_ on the membrane of Neuro-2a cells. *R_av_* is a parameter characterizing the formation of complexes between HgTx-G and Kv(1.1m-1.2)_2_.

Comparative analysis of the distribution of K-Kv1.1m, K-Kv1.1m-1.2, K-Kv1.1, and K-Kv1.1-1.2 constructs expressed in Neuro-2a cells was performed with confocal microscopy (Figure 1). K-Kv1.1m showed a web-like distribution in the cytoplasm of cells without noticeable accumulation at the plasma membrane (Figure 1a). This pattern was formed 15–17 h after transfection and remained almost unchanged on the second day after cell transfection. In contrast, K-Kv1.1m-1.2 demonstrated both a web-like cytoplasmic distribution and considerable staining of the plasma membrane starting from the first day after transfection (Figure 1b). Cellular distributions of K-Kv1.1-1.2 (Figure 1d) and K-Kv1.1 (Figure 1c) were very similar to that of K-Kv1.1m-1.2 (Figure 1b), but they formed on the first and second day after transfection, respectively.

HgTx-G, a fluorescent high-affinity ligand for homotetrameric channels Kv1.1 and Kv1.2 [35] and heterochannels Kv(1.1-1.2)_2_ [32], stained the membrane of cells expressing K-Kv1.1m-1.2 (Figure 1b) and (as it should be) K-Kv1.1 (Figure 1c) and K-Kv1.1-1.2 (Figure 1d), but did not bind to the cells expressing K-Kv1.1m (Figure 1a). The results suggest that K-Kv1.1m-1.2 accumulates in the membrane in the form of heterochannels Kv(1.1m-1.2)_2_, which retain affinity for HgTx-G. The lack of HgTx-G binding on the membrane of cells expressing K-Kv1.1m is consistent with the absence of K-Kv1.1m homotetramers in the plasma membrane.

According to the analysis of the concentration-dependent binding of HgTx-G to heterochannels Kv(1.1m-1.2)_2_ (Figure 1e), the dissociation constant of the complex is 170 ± 70 pM.

Thus, the T226R mutation blocks the membrane presentation of homotetrameric Kv1.1m channels but not the presentation of heterochannels Kv(1.1m-1.2)_2_.

### 2.3. Interactions Between α-Subunits Kv1.2 and Kv1.1m in Cells

α-Subunits Kv1.1 and Kv1.2 possess a natural property to form heterochannels [20]. Since mutations can potentially affect this property, we have studied the ability of α-subunits Kv1.2 and Kv1.1m to form complexes in Neuro-2a cells using FRET microscopy. To do this, the α-subunits Kv1.2 and Kv1.1m were co-expressed in cells as chimeric proteins C-Kv1.2 and K-Kv1.1m. Fluorescent proteins TagCFP and mKate2, which are present in these chimeras, are a donor-acceptor pair that can participate in FRET according to the analysis performed using the FRET calculator plugin at www.fpbase.org.

**Figure 2:**
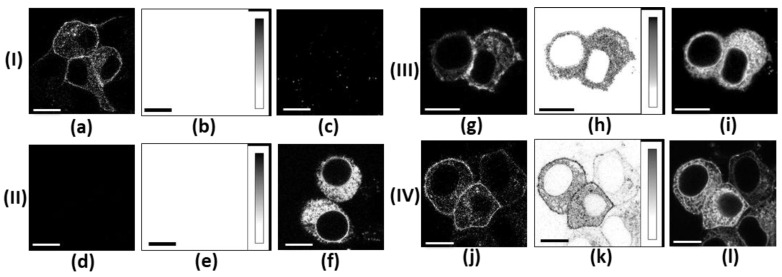
Confocal fluorescent images of cells expressing C-Kv1.2 (**I**), K-Kv1.1m (**II**), and C-Kv1.2 together with K-Kv1.1m (**III**,**IV**) at 24 h (**III**) or 48 h (**I**,**II**,**IV**) after transfection. (**a**,**d**,**g**,**j**) Images were recorded in the 465–525 nm range at λ_ex_ = 458 nm. They show distribution of C-Kv1.2 (**a**,**g**,**j**) or absence of fluorescence in cells expressing K-Kv1.1m at these imaging parameters (**d**). (**b**,**e**,**h**,**k**) Images were recorded in the 590–750 nm range at λ_ex_ = 458 nm and treated as described in Section 4.5 to compensate for cross-talk between fluorescence signals. They show the distribution of the FRET signal, i.e., heteromeric complexes between C-Kv1.2 and K-Kv1.1m (**h**,**k**), or the absence of a residual signal in the images of cells expressing C-Kv1.2 (**b**) or K-Kv1.1m (**e**) after the cross-talk compensation. (**c**,**f,i**,**l**) Images were recorded in the 590–750 nm range at λ_ex_ = 561 nm. They show the distribution of K-Kv1.1m (**f,i**,**l**) or a typical distribution of intrinsic cellular fluorescence at these imaging parameters in cells expressing C-Kv1.2 (**c**). The scale bar is 10 µm. The intensity scale in panels (**b**,**e**,**h**,**k**) has been inverted for clarity.

In our FRET experiments, the ranges of detection for the fluorescence of TagCFP and mKate2 were selected such that the contribution of TagCFP fluorescence to the detection range of mKate2 fluorescence and the direct excitation of mKate2 at the 458 nm excitation wavelength (λ_ex_ = 458 nm) were low. These contributions were carefully subtracted from the FRET signal using the measured cross-talk coefficients (Figure 2b,e,h,k). Intrinsic cellular fluorescence, which appeared as randomly distributed granules in the cytoplasm of cells, was weak at the selected imaging settings (Figure 2b,c,d,e). In cells co-expressing C-Kv1.2 and K-Kv1.1m, the implemented approach provides reliable imaging of the FRET signal, which indicates the formation of complexes between C-Kv1.2 and K-Kv1.1m (Figure 2h,k).

In 24 h after co-transfection, the complexes between C-Kv1.2 and K-Kv1.1m, as revealed by FRET imaging, were rather evenly distributed throughout the cytoplasm of all cells expressing both proteins (Figure 2h), while at the plasma membrane, these complexes were reproducibly observed in 5–10% of such cells. At the same time, the membrane localization of C-Kv1.2 alone was detected in many cells (Figure 2g), indicating the formation of homotetrameric channels Kv1.2 in addition to heterochannels Kv1.1m-Kv1.2. In 48 h after co-transfection, the portion of successfully transfected cells with the membrane localization of heterochannels increased to 20–25% (Figure 2k). We re-analyzed our previous FRET experiments with unmutated Kv1.1 and Kv1.2 α-subunits [34] and estimated that the portion of successfully transfected cells with the membrane presentation of unmutated heterochannels achieved 30–35% in 24 h after co-transfection. These data suggest efficient complexation between α-subunits Kv1.2 and Kv1.1(T226R) but a slower transfer of heterochannels Kv1.1m-Kv1.2 to the plasma membrane compared to homotetrameric channels Kv1.2 and heterochannels Kv1.1-Kv1.2. Considering the absence of membrane presentation of homotetrameric channels Kv1.1.m even 48 h after transfection (Figure 2f), the membrane-associated fluorescence of K-Kv1.1m in co-transfected cells at the direct excitation of mKate2 (λ_ex_ = 561 nm) confirms the membrane expression of heterochannels Kv1.1m-Kv1.2 and shows their widespread distribution in the plasma membrane (Figure 2l).

### 2.4. Electrophysiological Properties of Heterochannels Kv(1.1m-1.2)_2_

**Figure 3:**
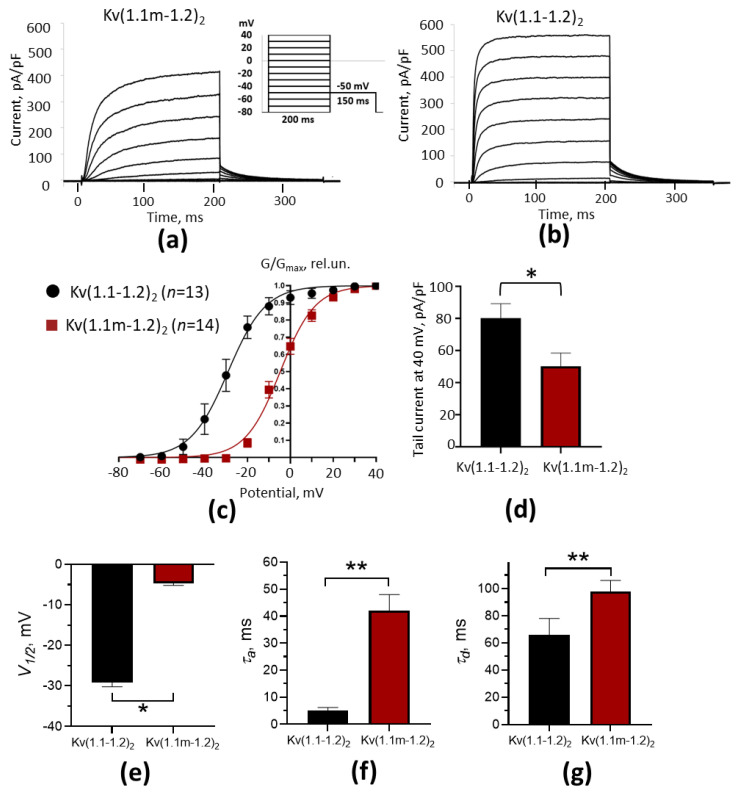
Comparison of electrophysiological properties of heterochannels Kv(1.1m-1.2)_2_ and Kv(1.1-1.2)_2_ in Neuro-2a cells. (**a**,**b**) Representative traces of currents recorded in cells expressing heterochannels Kv(1.1m-1.2)_2_ (**a**) and Kv(1.1-1.2)_2_ (**b**). Currents were induced using the square-pulse depolarization protocol (inset in panel a) from the holding potential of −80 mV. (**c**) Steady-state activation curves of heterochannels Kv(1.1-1.2)_2_ and Kv(1.1m-1.2)_2_ formed in Neuro-2a cells. G/G_max_ is the average normalized conductance of cells. (**d**) Densities of peak tail currents recorded after depolarization to 40 mV in cells expressing heterochannels Kv(1.1-1.2)_2_ and Kv(1.1m-1.2)_2_. (**e**) Half-maximum activation potentials. (**f**,**g**) Constants of channel activation (**f**) and deactivation (**g**). Significance of differences, *t*-test, *n* = (11÷13): * *p* < 0.05, ** *p* < 0.0001.

The whole-cell patch-clamp measurements in cells expressing K-Kv1.1m-1.2 show that this concatenated protein forms functional voltage-gated potassium channels in the plasma membrane (Figure 3a). The experiments that were conducted in parallel on cells expressing either K-Kv1.1m-1.2 or K-Kv1.1-1.2 revealed considerable differences in the properties of channels Kv(1.1m-1.2)_2_ and Kv(1.1-1.2)_2_ (Figure 3). Channels Kv(1.1m-1.2)_2_ have significantly slower kinetics of activation (τ_a_) and deactivation (τ_d_), and their activation occurs at a much less negative membrane potential compared to channels Kv(1.1-1.2)_2_ (Figure 3a–c, Table 1). Another difference is the current density in cells expressing the studied heterochannels. The tail current measured after depolarization of the cell membrane to 40 mV was approximately 1.6 times higher in the cells expressing Kv(1.1-1.2)_2_ channels than in the cells with Kv(1.1m-1.2)_2_ channels (Figure 3a,b,d). Considering that the mutation T226R negatively regulates the membrane presentation of homotetrameric channels Kv1.1 (Figure 1a), the reduced current in the cells with channels Kv(1.1m-1.2)_2_ is probably related to a decreased membrane presentation of these channels compared to unmutated channels Kv(1.1-1.2)_2_.

## 3. Discussion

Previous studies revealed that mutation T226R strongly decreased current through the depolarized membrane when the mutated Kv1.1 α-subunit was expressed in Xenopus laevis oocytes instead of the wild type (wt) α-subunit [24,30]. It was concluded that impaired trafficking to the membrane, rather than distorted conductance or decreased opening possibility of channels, was the reason for this [30]. Our fluorescence microscopy data show that, in mammalian cells, neither assembled homotetrameric channels Kv1.1 nor separate α-subunits reach the plasma membrane if they contain mutation T226R (Figure 1a). A negative effect of mutation T226R overcomes the membrane-targeting action of mutation S369T, specially introduced in the K-Kv1.1 protein (Figure 1a,c). This suggests that mutation T226R further reduces the natural very low probability of homotetrameric Kv1.1 channel presentation on the membrane of mammalian cells [33,36,37,38].

Heterochannels Kv1.1 formed by wt and mutated α-subunits in oocytes were shown to have currents that are intermediate between those through homotetrameric wt and mutant Kv1.1 channels [24,30]. This was considered to be a partial recovery of the membrane presentation of the mutant channels due to the presence of wt α-subunits. Mutation T266R in such heterochannels induced a shift in the channel activation to a less negative membrane potential and slowed down the kinetics of their activation and deactivation. It was concluded that such characteristics of Kv1.1 heterochannels carrying the T226R mutation can be associated with channel malfunction leading to seizures at a heterozygous phenotype [24,30]. At the same time, according to systematic data on the composition of Kv1 homo- and hetero-channels, the channels composed solely of Kv1.1 α-subunits have not been found in the mammalian brain, except for their occasional presence in non-myelinated axons at the periphery [20]. Since Kv1.1 α-subunits readily assemble with Kv1.2 α-subunits in the brain, heterochannels Kv1.1(T226R)-Kv1.2 should be considered as those that are involved in the development of disease symptoms. Unmutated heterochannels Kv(1.1-1.2)_2_ were found to have functional characteristics that are very similar to those of homotetrameric Kv1.1 channels [32]. This suggests that Kv1.1 α-subunits essentially define the properties of heterochannels Kv1.1-Kv1.2. In agreement with this supposition, mutation T226R considerably affected the properties of heterochannels Kv(1.1m-1.2)_2_ by shifting channel activation to a less negative membrane potential by 24 mV and decreasing the activation and deactivation rates by factors of 8 and 1.5, respectively, compared to those of unmutated heterochannels Kv(1.1-1.2)_2_ (Table 1). According to the obtained data, mutation T226R does not prevent the complexation between Kv1.1(T226R) and Kv1.2 α-subunits (Figure 2), but it does delay and possibly decrease the presentation of heterochannels in the plasma membrane (Figure 2h,k and Figure 3d). The unique properties of the Kv1.1 channel, which determine the low threshold and accelerated onset rate of K+ currents, are compromised by mutation T226R, and the presence of the Kv1.2 α-subunits in heterotetrameric channels does not compensate for the loss of these activities. The data obtained support the conclusion that mutation T226R in heterochannels decreases and delays the responsiveness of cells to mild membrane depolarization.

The dissociation constant of the HgTx-G complex with heterochannels Kv(1.1m-1.2)_2_, 170 ± 70 pM (Figure 1e), was found to be the same as the dissociation constant with heterochannels Kv(1.1-1.2)_2_, 100 ± 20 pM [32]. This suggests that mutation T226R does not affect the conformation of the binding site for the peptide blockers located at the outer vestibule of the channel pore. In turn, this makes it impossible to use peptide blockers (including fluorescently labeled peptides) for the detection and imaging of mutated heterochannels in cells.

Concerning physiology, the T226R mutation in heterochannels Kv1.1-Kv1.2 is most likely to disrupt the regulation of action potential propagation along the axon and impair the repolarization of the plasma membrane between action potentials, leading to excessive excitation of neurons. At the T226R phenotype, heterochannels Kv1.1(T226R)-Kv1.2 are possibly a key marker for the prognosis of emerging neurological disorders and may be a target for therapeutic intervention.

Our study was carried out on heterochannels with 2:2 stoichiometry. Obviously, this effect may depend on the number of mutant Kv1.1 α-subunits in the heterochannel. However, the distribution of heterochannels Kv1.1-Kv1.2 according to the stoichiometry of their constituent subunits in the brain remains unknown. Additionally, it is possible that heterochannels Kv1.1-Kv1.2 include both mutant and non-mutant Kv1.1 α-subunits expressed due to heterozygosity. This may further affect the properties of heterochannels carrying the mutation and require detailed investigation.

## 4. Materials and Methods

### 4.1. Reagents

GenJector-U transfection reagent was from Molecta (Moscow, Russia). HgTx-G was produced as described previously [35]. The concentration of HgTx-G in an aqueous solution was determined using the molar extinction coefficient for eGFP (56,000 M^−1^ cm^−1^ at 489 nm).

### 4.2. Construction of Expression Plasmids

Plasmids pmKate2-KCNA1, pmTagCFP-KCNA2, and pmKate2-KCNA1-KCNA2, which encode K-Kv1.1, TagCFP-Kv1.2, and the K-Kv1.1-1.2 dimeric concatemer, respectively, were obtained as described previously [32,33,34].

To insert the T226R mutation in the Kv1.1 α-subunit, a single nucleotide substitution was introduced in the *KCNA1* gene to change a codon ACG (T226) to AGG (R226). Site-directed mutagenesis was carried out by the overlap-extension polymerase chain reaction using the pmKate2-KCNA1 plasmid as a template, a pair of terminal primers SacI-f and BsteII-r, and a pair of mutagenic primers with overlapping sequences, R226-f and R226-r:

SacI-f—5′-CTCTTCGAGTACCCCGAGAGCTC-3′;

BsteII-r—5′-GTATCCTACAGTGGTCATGGTCACC-3′;

R226-f—5′-CATCGTGGAA**AGG**CTGTGTATCATC-3′;

R226-r—5′-GATGATACACAG**CCT**TTCCACGATG-3′.

Restriction enzyme sites SacI and BstEII used for cloning are underlined. The codon for R226 is marked in bold in the R226-f and R226-r primer**s**. The amplified 600 bp DNA fragment was digested with restriction endonucleases SacI and BstEII and cloned into the same sites of the plasmid pmKate2-KCNA1 to get pmKate2-KCNA1(T226R), which coded for the mutant α-subunit K-Kv1.1m. To construct concatenated dimer K-Kv1.1m-1.2, the gene *KCNA1(T226R)* from the plasmid pmKate2-KCNA1(T226R) was amplified in PCR using primers Kcna1-f1 (5′-TTCTCAGATCTATGACGGTGATGTCTGGGGAGAACGT-3′) and Kcna1-Stop-r1 (5′-TCTTCAAGCTTAACATCGGTCAGTAGCTTGC-3′) [32] and cloned into BglII/HindIII sites of the pmKate2-KCNA1-KCNA2 plasmid to obtain the pmKate2-KCNA1(T226R)-KCNA2 plasmid. The nucleotide sequence of the *KCNA1(T226R)* gene in pmKate2-KCNA1(T226R) and pmKate2-KCNA1(T226R)-KCNA2 was confirmed by Sanger sequencing (Evrogen, Moscow, Russia).

### 4.3. Cell Culture and Transfection

Mouse neuroblastoma Neuro-2a cells (the Institute of Cytology RAS, Saint Petersburg, Russia) were cultured as described previously [33,35]. Cells were seeded into 24-well plates (30,000 cells per well, 1 mL) on circular glass coverslips (10 mm, Gerhard Menzel GmbH, Braunschweig, Germany) precoated with poly-L-lysine (Paneco, Moscow, Russia). Transfection of cells was performed using GenJector-U reagent according to the manufacturer’s protocol at 30–40% cell confluence. The experiments were conducted 1 or 2 days after transfection. To analyze the binding of channels Kv(1.1m-1.2)_2_ with the fluorescent ligand, HgTx-G (0.02–1 nM) was added to cells in a complete medium for 1 h.

### 4.4. Electrophysiology Measurements

The whole-cell voltage clamp recording of ion currents was performed using an Axopatch 200A amplifier (Molecular Devices, Sunnyvale, CA, USA). All experiments were carried out at 23 ± 1 °C. Cover glasses with Neuro-2a cells were placed into the chamber with a continuous flow of K^+^-based external saline solution containing (in mM): 150 NaCl, 5.4 KCl, 1.8 CaCl_2_, 1.2 MgCl_2_, 10 glucose, and 10 HEPES (pH 7.4). The selection of cells for measurement was based on intense red fluorescence as an indicator of successful transfection. Patch pipettes of 1.5–2.5 MΩ resistance were pulled from borosilicate glass (Sutter Instrument, Novato, CA, USA) and filled with K^+^-based electrode solution containing (in mM): 140 KCl, 1 MgCl_2_, 5 EGTA, 4 MgATP, and 10 HEPES (pH 7.2). Series resistance and capacitance of the pipette and cell were routinely compensated. Current amplitudes were normalized to the capacitive cell size.

To obtain *I*-*V* curves and steady-state activation curves, the current passing through heterochannels was measured every 2 s starting from the holding potential (−80 mV) using a standard double-pulse protocol (Figure 3a, insert). The initial 200 ms depolarization of membrane from −80 to +40 mV (in 10 mV steps) was followed by a 150 ms repolarization to −50 mV, allowing tail currents to be recorded. The peak tail current values were used to calculate the normalized conductivity ratio (*G/G_max_*, where *G_max_* is the maximum conductivity). The dependence of *G/G_max_* on the applied potential *V* was fitted to the Boltzmann equation in order to determine the half-maximum activation potential (*V*_1/2_):

*G*/*G_m_* = 1/(1 + exp((*V*_1/2_−*V*)/*k*)) (1)

where *k* is the slope coefficient.

*V*_1/2_ was averaged over measured cells and presented as mean ± SEM.

The values of *τ*, which characterize the kinetics of channel activation and deactivation, were calculated using a monoexponential equation for each of the activation and tail current traces measured. The dependences of τ on the applied potential were plotted, and the constants τ_x_ at *V*_1/2_ were determined for channel activation (τ_a_) and deactivation (τ_d_) using a general equation:

τ = τ_x_ exp((*V*−*V*_1/2_)/*s*) (2)

where *s* is the slope of the τ-*V* dependence curve.

Values of τ_a_ and τ_d_ were averaged over measured cells and presented as mean ± SEM.

The Kolmogorov–Smirnov test was used to check whether data (*V*_1/2***,***_ τ_a_, and τ_d_) fit the normal distribution. The parameters of a current in different groups of cells were compared using an uncoupled *t*-test. *p* values of <0.05 were considered statistically significant.

### 4.5. Confocal Microscopy

Microscopy was carried out using the SP2 confocal microscope with the HCX PL APO 63×/1.2 objective (Leica Microsystems GmbH, Wetzlar, Germany). The size of the confocal diaphragm corresponded to 1 Airy disk. The typical voxel size was 0.23 × 0.23 × 0.9 μm. The fluorescent imaging was performed as follows: λ_exc_ = 561 nm, detection in the 650–700 nm range for the mKate2-related signals; λ_exc_ = 488 nm, detection in the 498–535 nm range for HgTx-G. To avoid any cross-talk between signals in two-color experiments, sequential scanning was used.

The interaction of HgTx-G with channels Kv(1.1m-1.2)_2_ was analyzed as described earlier [33,39]. Briefly, the ratio (*R*) of the fluorescence intensities of HgTx-G and channels Kv(1.1m-1.2)_2_ at the plasma membrane of a cell was calculated and averaged over the measured cells (22–32 cells) to obtain the *R_av_* value and its standard deviation. The dependence of *R_av_* on the HgTx-G concentration (*L*) was fitted with the equation

*R_av_*(*L*)/*R_m_* = *L*/(*K_d_* + *L*) (3)

where *K_d_* is the dissociation constant of the complex and *R_m_* is the maximal *R_av_* value. *K_d_* was determined in three independent measurements and presented as mean ± SEM.

In FRET experiments, APD detectors were used for confocal imaging. The fluorescence of CFP-Kv1.2 was excited at 458 nm and recorded in the 465–525 nm range. The fluorescence of K-Kv1.1m was excited at 561 nm and detected in the 590–750 nm range. To measure the FRET signal, fluorescence was excited at 458 nm and recorded in the 590–750 nm range. The contribution of TagCFP fluorescence to the detection range of mKate2 fluorescence was 10.5 ± 0.2%. The intensity of mKate2 fluorescence in the range of 590–750 nm at λ_ex_ = 458 nm was 2.5 ± 0.3% of the corresponding intensity at λ_ex_ = 561 nm. These two cross-talk signals were subtracted from the images corresponding to the FRET signal.

## 5. Conclusions

The results of the present study show that the mutation T226R in the Kv1.1 α-subunit significantly affects the activation and deactivation of heterochannels Kv1.1-Kv1.2. Considering that Kv1.1 α-subunits readily assemble with Kv1.2 α-subunits in the brain, heterochannels Kv1.1-Kv1.2 bearing the T226R mutation are likely responsible for the malfunctioning of the ensemble of ion channels at EA1 [24,28], neuromyotonia [28], developmental delay, and periodic muscle spasms [29]. Based on the data on the distribution of Kv1.1 α-subunits in the brain [20], one may suppose that synaptic transmission and neuronal activity are essentially affected by aberrant functioning of heterochannels Kv1.1(T226R)-Kv1.2.

## Figures and Tables

**Table 1:** Electrophysiological characteristics of heterochannels Kv(1.1-1.2)_2_ and Kv(1.1m-1.2)_2_ in Neuro-2a cells.

Channels	*V*_1/2_ ^¶^, mV	τ_a_, ms	τ_d_, ms
Kv(1.1-1.2)_2_	−29.0 ± 1.1 (*n* = 13)	5.1 ± 1.1 (*n* = 11)	66 ± 12 (*n* = 11)
Kv(1.1m-1.2)_2_	−4.7 ± 0.5 (*n* = 14), *p* * < 0.0001	42 ± 6 (*n* = 11), *p* * < 0.0001	98 ± 8 (*n* = 11), *p* * = 0.038

The data are mean ± SEM, *n* is the number of studied cells; ^¶^ is the half-maximum activation potential; and * is the significance of the difference between heterochannels Kv(1.1m-1.2)_2_ and Kv(1.1-1.2)_2_ that was determined using an uncoupled *t*-test.

## Data Availability

The data presented in this study are available on request from the corresponding author. The data are not publicly available due to local regulations.

## Abbreviations

The following abbreviations are used in this manuscript:

EA1	episodic ataxia type 1
FRET	Förster resonance energy transfer
